# Effect of the modified RADAR, No-touch, and conventional techniques on arteriovenous fistula outcomes

**DOI:** 10.1080/0886022X.2026.2679808

**Published:** 2026-06-02

**Authors:** Fan Zhang, Lihong Zhang, Rui Cui, Xiangru Li, Jing Wen, Fang Hou, Xibin Hou, Shanshan Guo, Yi Zeng, Yuzhu Wang, Shen Zhan

**Affiliations:** Department of Nephrology, Haidian Hospital (Haidian Section of Peking University Third Hospital), Beijing, China

**Keywords:** Arteriovenous fistula, modified RADAR technique, primary patency, juxta-anastomotic stenosis, maturation, no-touch technique

## Abstract

**Objective:**

Low primary patency rates represent a major problem of radiocephalic arteriovenous fistula (RC-AVF). No-touch techniques and radial artery deviation and reimplantation (RADAR) were reported and used in RC-AVF creation. To enhance RC-AVF outcomes, we devised a modified RADAR technique and conducted a retrospective study comparing this approach with no-touch and conventional methods for RC-AVF creation.

**Methods:**

We retrospectively reviewed patients undergoing RC-AVF creation for hemodialysis using either the modified RADAR, no-touch, or conventional technique between January 2023 and December 2024. The primary patency, juxta-anastomotic stenosis, and maturation rates of the three techniques were compared and analyzed.

**Results:**

In total, 289 patients were included. The incidence of anastomotic stenosis was significantly lower (*p* = 0.030) in the modified RADAR group (33.33%) than in the no-touch technique (60.32%) and conventional technique groups (50.40%). The AVF maturation rate within 6–8 weeks was significantly higher (*p* = 0.005) in the modified RADAR group (90.70%) than in the no-touch technique (67.06%) and conventional technique groups (65.84%). At 3, 6, 9 and 12 months postoperatively, the primary patency rates in the modified RADAR group were 90.7%,88.37%, 86.05% and 86.05%, respectively. Significant differences were observed among the three groups (log-rank *p* = 0.04).

**Conclusion:**

The modified RADAR technique for RC-AVF creation resulted in higher primary patency, lower juxta-anastomotic stenosis, and improved maturation rates.

## KEY MESSAGES

1. **What is known:** Low primary patency rates represent a major problem of radiocephalic arteriovenous fistula.
2. **This Study add:** This retrospective study of 289 patients demonstrates that the modified RADAR technique significantly reduces juxta-anastomotic stenosis (33.33% vs. 50.40–60.32%) and achieves a 12‑month primary patency rate of 86.05%.
3. **Potential Impact:** Compared with the no-touch and conventional techniques, RC-AVF creation by the modified RADAR technique decreases juxta-anastomotic stenosis, improves AVF maturation, and increases primary patency.

## LAY SUMMARY

For patients undergoing hemodialysis, establishing a reliable vascular access – most commonly a radiocephalic arteriovenous fistula (RC-AVF) – is critical. However, many fistulas either fail to mature properly or become narrowed (stenosis) over time, which limits their effectiveness for dialysis.

In this study, we developed a modified RADAR surgical technique in RC-AVF creation. We compared this new technique against the standard no-touch method and conventional technique in 289 patients who underwent RC-AVF creation between January 2023 and December 2024.

Our results showed that the modified RADAR technique significantly improved maturation rates (90.7% vs. 65–67%) and reduced juxta-anastomotic stenosis(33.3% vs. 60.3% for no-touch and 50.4% for conventional techniques). Furthermore, primary patency rates at 3, 6, 9, and 12 months were highest in the modified RADAR group (90.7%, 88.4%, 86.1%, and 86.1%, respectively), with statistically significant differences across all three groups.

These findings suggest that the modified RADAR technique offers a promising alternative for RC-AVF creation, potentially reducing the need for repeat interventions and improving long-term outcomes for patients on hemodialysis.

## Graphical Abstract

**Figure:**
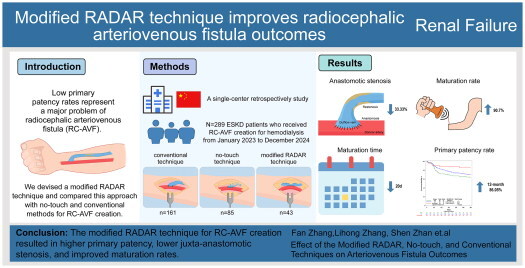

## Introduction

Hemodialysis serves as a life-sustaining treatment for patients with end-stage renal disease, and the creation of functional vascular access is a critical component of this therapy. Currently, an autogenous radiocephalic arteriovenous fistula (RC-AVF) is recommended as the preferred vascular access whenever possible [1]. Nonetheless, the dysfunction rate of conventional RC-AVFs, including complications such as immaturity, juxta-anastomotic stenosis, and thrombosis, remains high [2]. Many factors contribute to AVF dysfunction, including sex, age, underlying disease, vessel diameter, and surgical technique. Concerning the surgical technique, prior studies reported modified techniques for AVF creation. For example, researchers employing the ‘no-touch’ technique during AVF creation – where the vein is dissected with preservation of perivascular adipose tissue – observed superior maturation and patency rates compared to conventional technique in patients with small veins [3,4]. Sadaghianloo et al. [5] further explored no-touch technology and proposed the radial artery deviation and reimplantation (RADAR) technique, which transposes the radial artery onto the cephalic vein to create a radial-cephalic AVF without venous dissection [6]. This technique has been reported to cause less juxta-anastomotic stenosis and achieve better maturation and patency than standard radiocephalic fistula creation techniques [5]. However, after ligation of the distal artery at the anastomosis site, the future creation of an AVF or arteriovenous graft (AVG) at the elbow could be hampered, potentially leading to hand ischemia.

To further minimize intra-operative vessel injury, we developed a modified RADAR technique that that preserves the benefits of the original approach while lowering the risk of juxta-anastomotic venous stenosis [7]. This is achieved by preserving the integrity and patency of the radial artery, attributable to our modification of the RADAR procedure, which transitioned from an end-to-side anastomosis between the radial artery and cephalic vein to a side-to-side anastomosis. In this study, we assessed the efficacy of the modified RADAR, no-touch, and conventional techniques concerning RC-AVF outcomes.

## Materials and methods

### Study design and patients

We retrospectively analyzed ESKD patients who received RC-AVF creation for hemodialysis at the Nephrology Department of Beijing Haidian Hospital from January 2023 to December 2024. During this period, the conventional technique, no-touch technique, and modified RADAR were adopted consecutively. The inclusion criteria included no previous history of arteriovenous fistula surgery in the ipsilateral arm, radial artery diameter ⩾1.0 mm, cephalic vein diameter⩾2.0 mm, tourniquet use, and continuous upper arm vein run-off. The distance between the artery and vein was < 2 cm for patients in the modified RADAR group. The data were collected from their medical records at the time of admission. These data included demographic and clinical information such as age, sex, duration of hemodialysis, comorbidities, and medication history. In addition, clinical and biochemical parameters were measured, including complete blood count, albumin, triglycerides, calcium, phosphorus, intact parathyroid hormone and preoperative vascular lumen diameter. Postoperative ultrasound follow-up and patency-related incidence were also retrieved. Exclusion criteria included presence of ipsilateral central venous lesions, incomplete baseline data, or insufficient follow-up. All patients provided written informed consent. This study protocol was reviewed and approved by the Ethics Committee of Beijing Haidian Hospital (No.2024132). The study involving human participants adhered to ethical standards set by the institution and the national research committee. It was in accordance with the principles outlined in the 1964 Helsinki Declaration and its later amendments or similar ethical standards.

### Surgical procedures

**Figure 1.**
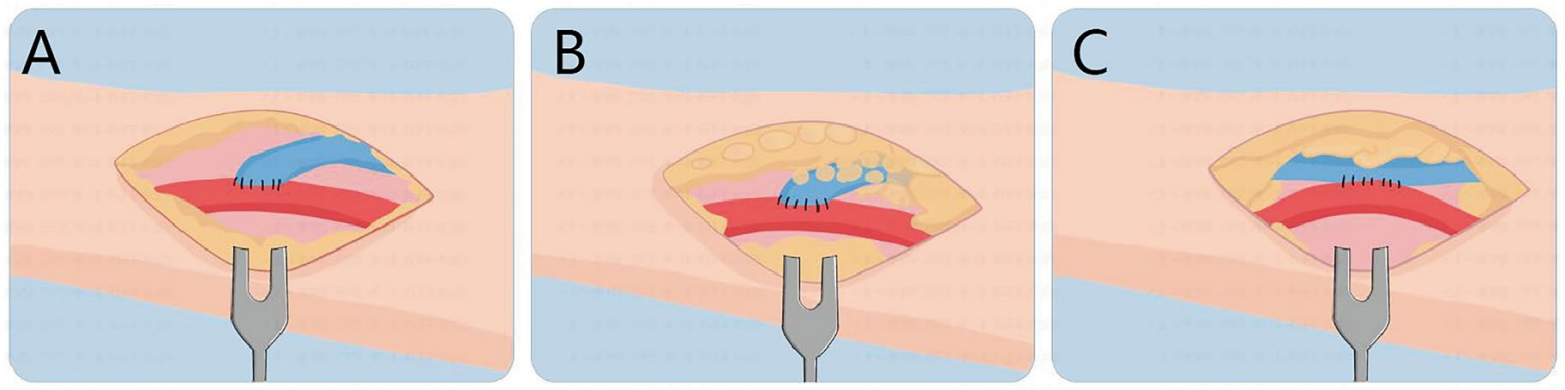
Schematic diagram of AVF: A. Conventional technique; B. No-touch technique; C. Modified RADAR technique.

**Figure 2.**
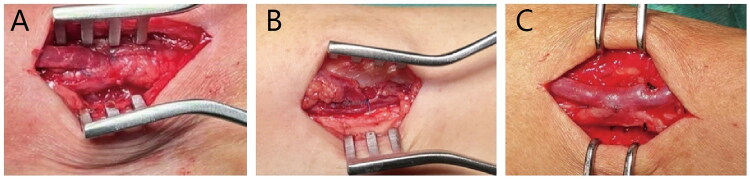
Intraoperative pictures: A. Conventional technique; B. No-touch technique; C. Modified RADAR technique.

The same surgeon (S.Z.) performed all operations. Peripheral nerve block anesthesia consisting of 1% lidocaine solution was used during the procedure. Through a 3–5 cm longitudinal skin incision placed on the forearm, an appropriate segment of the cephalic vein was harvested using either the conventional or no-touch technique. In the conventional technique group, the perivascular adipose tissue of the cephalic vein was removed (Figure 1(A) and 2(A)). In the no-touch technique group, the vein was gently mobilized with deliberate preservation of the perivascular adipose tissue (Figure 1(B) and 2(B)) [4]. Then the radial artery was exposed through a 3–5 cm perivascular tissue dissection and an 8–10 mm longitudinal incision was created with flow occluded. Subsequently, the distal end of the cephalic vein was anastomosed to the radial artery in an end-to-side fashion.

In the modified RADAR group, a longitudinal skin incision of approximately 5 cm was made along the forearm between the radial artery and cephalic vein. Blunt dissection with curved hemostats was employed to gently separate the adipose tissue until the superficial fascia became visible. After exposing the cephalic vein within the superficial fascia, the superficial fascia was preserved. The vein was virtually untouched, thereby preserving its perivascular tissue and nutrient vessels. Bulldog forceps were applied on both sides of the cephalic vein to occlude venous blood flow en bloc with the surrounding perivenous tissue. The radial artery was mobilized along a sufficient length to permit repositioning adjacent to the vein. With blood flow occluded in both vessels, side-to-side anastomosis was constructed (Figure 1(C) and 2(C)). A running 7‑0 polypropylene suture was used for all anastomoses.

### Ultrasound examination protocols

All ultrasound examinations were performed using a high-frequency linear transducer (7–20 MHz).

#### Preoperative assessment

Patients were positioned supine with the upper arm exposed at room temperature. The following parameters were assessed: (1) radial artery diameter, peak systolic velocity (PSV), and presence of calcification or stenosis at the proposed anastomosis site; (2) cephalic vein diameter at the proposed anastomosis site in the forearm, compressibility, depth from skin, and presence of stenosis or large tributaries; (3) upper arm cephalic vein, axillary vein, and subclavian vein to rule out outflow stenosis.

#### Postoperative assessment

Ultrasound examinations were performed at 4, 6, and 10 weeks postoperatively. The following parameters were measured: (1) radial artery diameter 2 cm distal to the anastomosis; (2) outflow vein diameter 5 cm distal to the anastomosis and depth from skin; (3) anastomotic lumen diameter; (4) brachial artery blood flow volume; (5) for any detected stenoses, the location, minimum diameter, length, and distance from the anastomosis were documented.

### Outcomes

As of April 2026, the mean follow-up duration was 636 (205,937) days. The primary outcome was primary patency, defined as the interval from AVF creation until the first occurrence of any intervention required to maintain or restore its patency or until abandonment or failure of the fistula without any prior corrective procedures [8]. Juxta-anastomotic stenosis was defined as *a* ≥ 50% reduction in the luminal diameter of the adjacent vessel segment accompanied by hemodynamic, functional, or clinical abnormalities that was not explained by reasons other than the dialysis vascular access lesion [9]. RC-AVF maturation was defined as an access flow of 600 mL/min with a vein at least 5 mm in diameter, as measured by duplex ultrasound [10,11].

### Statistical analysis

All data were analyzed using SAS 9.4 software (SAS Institute Inc., Cary, North Carolina) and GraphPad Prism 7.04 (GraphPad Software, Inc, San Diego, CA, USA). Continuous data are expressed as mean ± standard deviation and compared using the independent t-test (normal distribution) or Kruskal-Wallis test (non-normal distribution). Categorical variables are reported in frequency and percentage, and compared using the Chi-square test or Fisher’s exact Test. Juxta-anastomotic stenosis, AVF maturation rate and maturation time between the three groups were compared with Chi-square test and Kruskal–Wallis test. ANOVA was used to compare differences in postoperative venous diameter and blood flow among the groups during follow-up. Kaplan–Meier survival curves were plotted to assess patency, and the log-rank test was used to compare primary patency rates between the three groups. A two-tailed *p*-value less than 0.05 is statistically significant.

## Results

### Baseline demographic and clinical characteristics of patients in the three groups

The baseline demographic and clinical characteristics of the patients in the conventional technique, no-touch technique, and modified RADAR groups are summarized in Tables 1 and 2. In total, 161, 85, and 43 patients underwent surgery using the conventional technique, no-touch technique, and modified RADAR technique, respectively. Among them, 159 patients (55.02%) were male, and the mean patient age was 57.54 ± 13.3 years. Baseline characteristics including primary disease, age, height, dialysis duration, smoking history, and rate of comorbid hypertension or diabetes were not significantly different among the groups. The proportion of patients with comorbid cardiovascular disease and cerebrovascular disease was higher in the conventional technique group than in the no-touch technique and modified RADAR groups (Table 1).

**Table 1.** Characteristics of patients in conventional technique, no-touch technique and modified RADAR group.

Characteristic	Total *N* = 289	Conventional technique *N* = 161	No-touch technique *N* = 85	Modified RADAR *N* = 43	*p*-value
Age, years	57.54 ± 13.3	58.14 ± 13.46	55.74 ± 13.03	58.81 ± 13.16	0.324
Sex, n(%)					
Male	159 (55.02)	94 (58.39)	45 (52.94)	20 (46.51)	0.343
Female	130 (44.98)	67 (41.61)	40 (47.06)	23 (53.49)	.
Height, cm	1.67 ± 0.08	1.67 ± 0.08	1.67 ± 0.08	1.66 ± 0.08	0.915
BW, kg	65.7 ± 13.63	67.73 ± 14.32	63.78 ± 13.04	61.85 ± 10.69	0.013
BMI	23.6 ± 4.19	24.29 ± 4.41	22.91 ± 3.91	22.37 ± 3.36	0.006
SBP, mmHg	147.8 ± 13.73	148.88 ± 13.9	146.38 ± 14.0	146.53 ± 12.2	0.325
DBP, mmHg	82.42 ± 8.75	83.26 ± 8.99	82.96 ± 7.34	78.19 ± 9.38	0.002
Primary disease, n(%)					
Chronic nephritis	81 (28.03)	44 (27.33)	29 (34.12)	8 (18.60)	0.104
Diabetes nephropathy	96 (33.22)	56 (34.78)	28 (32.94)	12 (27.91)	.
Hypertension	92 (31.83)	53 (32.92)	23 (27.06)	16 (37.21)	
Others	20 (6.92)	8 (4.97)	5 (5.88)	7 (16.28)	.
Comorbidity					
Hypertension	268 (92.73)	149 (92.55)	77 (90.59)	42 (97.67)	0.342
Diabetes	140 (48.44)	86 (53.42)	38 (44.71)	16 (37.21)	0.120
Coronary heart disease	45 (15.57)	35 (21.74)	4 (4.71)	6 (13.95)	0.002
Cerebrovascular Disease	13 (4.5)	13 (8.07)	0 (0)	0 (0)	0.002
Smoking history, n(%)	7 (2.42)	3 (1.86)	4 (4.71)	0 (0)	0.293
Ipsilateral catheterization history, n(%)	44 (15.22)	28 (17.39)	14 (16.47)	2 (4.65)	0.110
Immunosuppressants, n(%)	6 (2.08)	2 (1.24)	4 (4.71)	0 (0)	0.187
Antiplatelet agents, n(%)	2 (0.69)	1 (0.62)	1 (1.18)	0 (0)	1.000

BW: Body weight; BMI: Body Mass Index; SBP: systolic blood pressure; DBP: diastolic blood pressure.

**Table 2.** Laboratory results of patients in conventional technique, no-touch technique and modified RADAR group.

Characteristic	Total *N* = 289	Conventional technique *N* = 161	No-touch technique *N* = 85	Modified RADAR *N* = 43	*p*-value
WBC	6.52 ± 2.36	6.77 ± 2.61	6.09 ± 1.92	6.46 ± 2.06	0.110
RBC	3.54 ± 0.72	3.58 ± 0.73	3.57 ± 0.68	3.34 ± 0.71	0.133
Hemoglobin	104.7 ± 20.4	105.76 ± 20.6	105.68 ± 20.3	98.98 ± 18.96	0.135
Platelet	198.02 ± 72.8	198.38 ± 71.8	196.41 ± 71.5	199.74 ± 80.3	0.967
C-reactive protein	1.99 (0.5, 4.67)	2.27 (0.58, 5.05)	2.02 (0.58, 3.51)	0.9 (0.5, 2.8)	0.076
Albumin	39.93 ± 5.37	39.92 ± 5.68	39.96 ± 4.91	39.91 ± 5.16	0.998
FPG	6.63 (5.32, 8.64)	6.82 (5.36, 8.7)	6.56 (5.04, 9.38)	6.1 (5.42, 7.24)	0.419
TG	1.6 (1.12, 2.29)	1.56 (1.08, 2.24)	1.54 (1.12, 2.22)	1.83 (1.3, 2.86)	0.101
K^+^	4.68 ± 0.73	4.6 ± 0.73	4.76 ± 0.72	4.77 ± 0.69	0.196
Ca	2.26 ± 0.24	2.27 ± 0.26	2.27 ± 0.21	2.22 ± 0.22	0.387
P	1.72 ± 0.57	1.71 ± 0.58	1.72 ± 0.62	1.76 ± 0.44	0.880
PTH	187 (93.4, 330)	153 (77.3, 289)	129 (88.1, 432)	223.5 (131, 369)	0.216
BNP	3704 (1254, 8873)	3832 (1201, 10022)	4070.5 (1254, 8404)	2679 (1661, 3697)	0.840
D-dimer	0.32 (0.2, 0.52)	0.32 (0.19, 0.54)	0.3 (0.21, 0.48)	0.38 (0.23, 0.72)	0.382
Cuffed venous diameter, mm	2.8 ± 0.6	2.8 ± 0.6	2.8 ± 0.6	2.8 ± 0.60	0.817
Uncuffed venous diameter, mm	2.1 ± 0.5	2.1 ± 0.5	2 ± 0.5	2.1 ± 0.5	0.635
Arterial lumen diameter, mm	2.0 ± 0.5	2.0 ± 0.6	1.9 ± 0.6	2.0 ± 0.4	0.560
Peak Radial Artery Velocity, cm/s	53 ± 17	54 ± 17	46 ± 17	55 ± 16	0.021

WBC: white blood cells; RBC: red blood cells; FPG: fasting plasma glucose; TG: triglyceride; K^+^: potassium; Ca: calcium; P: phosphorus; PTH: parathyroid hormone; BNP: B-type natriuretic peptide.

There were no significant differences in laboratory results among the three groups (Table 2). The preoperative diameters of the antecubital cephalic vein and radial artery did not differ among the groups. The radial artery peak velocity showed a statistically significant difference among the groups (*p* = 0.021), as detailed in Table 2.

### Postoperative ultrasound follow-up

**Figure 3.**
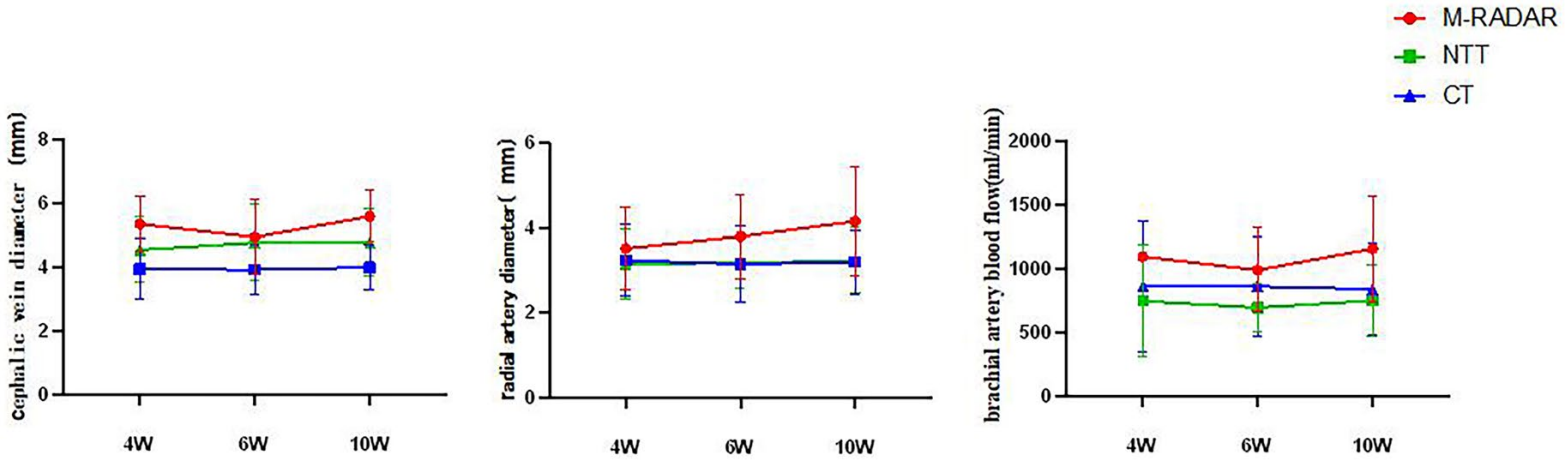
Postoperative ultrasound follow-up.

Follow-up ultrasound examination revealed uniform dilation of arteriovenous diameters and a gradual increase in brachial artery blood flow. At each postoperative time point, the modified RADAR group featured significantly larger venous diameters and greater blood flow than the other two groups (*p* < 0.05). Brachial artery blood flow was significantly higher (*p* = 0.001) in the modified RADAR group than in the no-touch technique and conventional technique groups (Table 3, Figure 3).

**Table 3.** Postoperative ultrasound follow-up.

Time	Value	Total	Conventional technique	No-touch technique	Modified RADAR	*p*-value
4W	anastomotic lumen diameter	6.0 ± 2.9	5.5 ± 2.2	4.5 ± 2.0	10 ± 2.7	<0.001
	venous diameter	4.5 ± 1.1	4.5 ± 1.0	4.0 ± 0.9	5.4 ± 0.9	<0.001
	arterial diameter	3.2 ± 0.9	3.2 ± 0.8	3.2 ± 0.8	3.5 ± 1.0	0.257
	brachial artery blood flow	867 ± 481	861 ± 513	744 ± 436	1092 ± 39	0.036
6W	anastomotic lumen diameter	5.7 ± 2.5	5.6 ± 1.8	4.4 ± 1.7	10.3 ± 3.4	<0.001
	venous diameter	4.5 ± 1.1	4.8 ± 1.2	3.9 ± 0.8	4.9 ± 1.2	0.013
	arterial diameter	3.2 ± 0.7	3.2 ± 0.6	3.2 ± 0.9	3.8 ± 1.0	0.098
	brachial artery blood flow	826 ± 351	859 ± 392	693 ± 192	987 ± 329	0.092
10W	anastomotic lumen diameter	6.1 ± 2.5	5.8 ± 1.8	5.0 ± 2.2	9.7 ± 2.8	<0.001
	venous diameter	4.7 ± 1.1	4.8 ± 1.1	4.0 ± 0.7	5.6 ± 0.8	<0.001
	arterial diameter	3.4 ± 0.9	3.2 ± 0.8	3.2 ± 0.8	4.1 ± 1.3	0.001
	brachial artery blood flow	857 ± 366	838 ± 355	747 ± 283	1153 ± 416	0.001

### Maturation rates and stenosis incidence

Within 6–8 weeks postoperatively, the modified RADAR group exhibited an anastomotic stenosis rate of 33.33%, which was lower than that in the no-touch technique and conventional technique groups (*p* = 0.030). The modified RADAR group achieved a maturation rate of 90.7% with a median maturation time of 20 (14, 35) days, demonstrating superior outcomes compared with those in the other two groups (Table 4).

**Table 4.** Maturation rates and incidence of stenosis during follow-up.

Value	Total	Conventional technique	No-touch technique	Modified RADAR	*p*-value
Juxta-anastomotic stenosis (%)	114 (50.22)	63 (50.4)	38 (60.32)	13 (33.33)	0.030
6–8W maturation (%)	202 (69.9)	106 (65.84)	57 (67.06)	39 (90.7)	0.005
Mature time(D)	35.5 (27, 59)	37 (24, 60)	42 (33, 60)	20 (14, 35)	<0.001

### Primary patency rates

**Figure 4.**
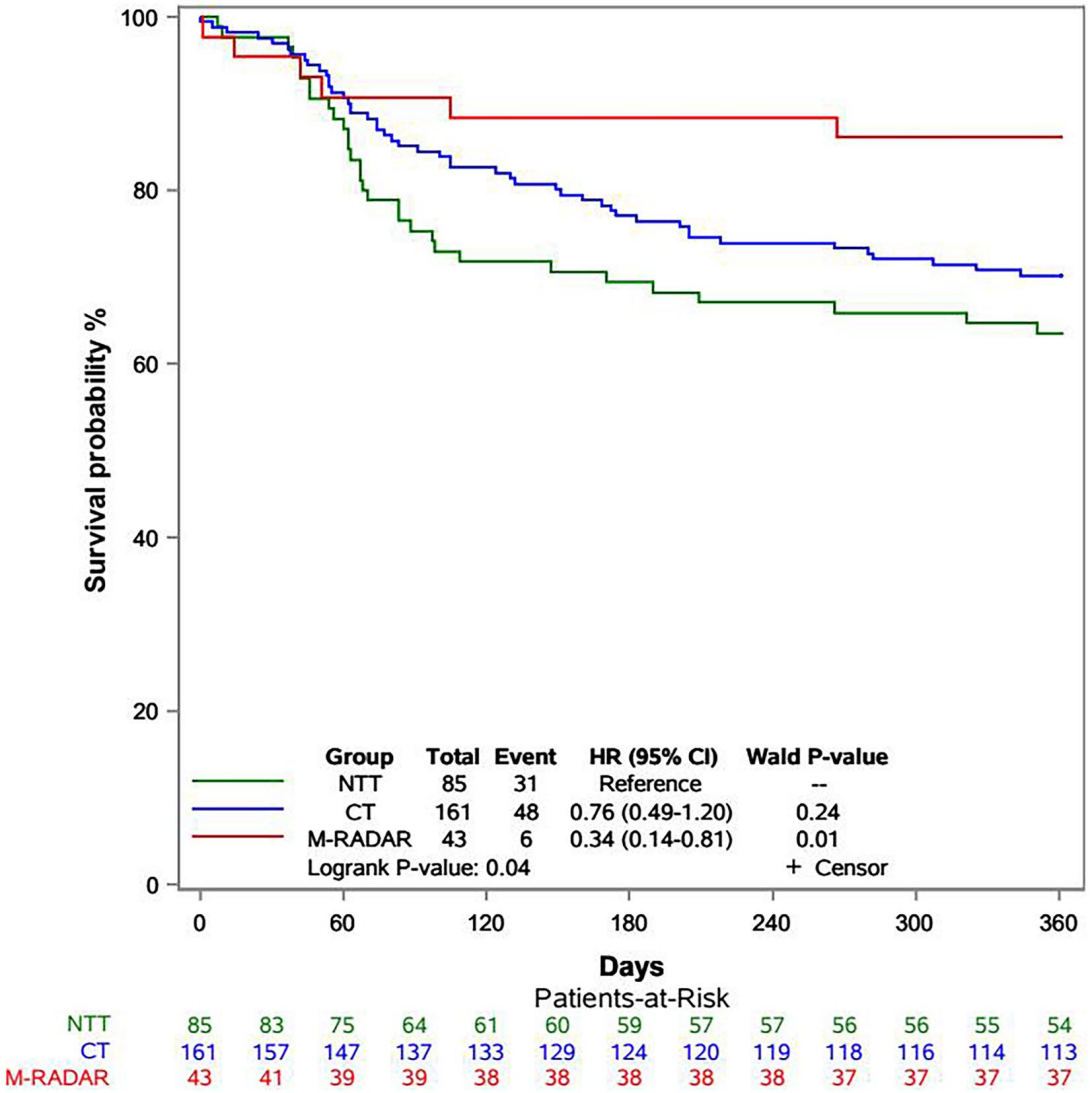
Primary patency analysis for comparison among conventional technique, no-touch technique and modified RADAR technique group.

Kaplan–Meier curve analysis of primary patency indicated that patients in the modified RADAR group had higher patency rates than the no-touch technique and conventional technique groups (*p* = 0.04, Figure 4). At 3, 6, 9 and 12 months postoperatively, the primary patency rates in the modified RADAR group were 90.7%, 88.37%, 86.05% and 86.05%, respectively. These rates were 75.29%, 69.41%, 65.88% and 63.53%, respectively, in the no-touch technique group and 85.09%, 77.02%, 73.29% and 70.19%, respectively, in the conventional technique group.

### Complication

During follow-up, two patients experienced reflux into the distal vein because of the absence of distal venous ligation. As the patients reported subjective discomfort, surgical ligation of the distal anastomotic vein was subsequently performed. No hand swelling occurred in any patients.

## Discussion

RC-AVF is a common option for achieving vascular access in patients on hemodialysis. However, the success rate of AVF maturation is approximately 50%–65% [12]. Therefore, maturation failure is common, and this complication usually results in negative outcomes including morbidity and mortality [13]. Juxta-anastomotic stenosis is among the main factors affecting the patency and maturation of RC-AVF [14].

There are various causes of maturation failure, including hemodynamic and mechanical stresses. Hemodynamic stress creates a turbulent flow that results in insufficient wall shear stress, leading to stenosis. Mechanical stress occurs because of excessive pressure and consequently causes vessel thickening. The combination of both stresses increases the risk of vessel stenosis and failure of fistula maturation [6]. In addition, recent studies found that traditional surgical dissection and mobilization of the vein disrupt the vasa vasorum, causing wall ischemia and oxidative stress [15]. Consequently, vascular cell migration and proliferation participate in venous wall thickening, causing neointimal hyperplasia that in turn can cause stenosis and AVF dysfunction [16,17]. Therefore, to address the above mechanisms, several previous studies have explored surgical techniques aimed at improving the maturation and patency rates of RC-AVF. The no-touch technique was applied to RC-AVF creation in 2016 [3]. In the AVF creation procedure, perivascular adipose tissue is preserved to reduce damage to the cephalic vein wall. Although this strategy did not drastically improve AVF patency, it offers the potential to create a RC-AVF in patients who are not usually considered appropriate for distal arm fistula creatin because of a small cephalic vein [3]. In 2015, Sadaghianloo et al. [4] described the RADAR technique. This technique extended the conventional no-touch technique and advocated the avoidance of any venous dissection. Through comparisons with retrospective control groups, studies reported a significant improvement in maturation with good primary patency rates during short- and long-term follow-up. However, ligation of the distal artery could preclude future AVF or AVG creation at the elbow, potentially leading to hand ischemia. In addition, the RADAR technique has been criticized by some authors [18]. Moreover, the RADAR technique cannot be used to create fistulas in the upper arm.

Based on the mechanism of maturation failure and juxta-anastomotic stenosis as well as related measures, we developed the modified RADAR technique. Our conceptual approach is derived from the RADAR technique: the vein is left completely *in situ*, and the artery is brought to the vein – essentially a RADAR variant. In 1966, Brescia et al. [19] described the first arteriovenous fistula, which also utilized a side-to-side anastomosis of the radial artery and cephalic vein. However, the geometry of the anastomosis and the hemodynamic profile in our modified RADAR technique differ from those of traditional side-to-side anastomosis. Compared to the conventional side-to-side anastomosis – where both the artery and vein are fully mobilized and approximated toward each other – our modified RADAR technique preserves the native course of the vein without mobilization. Instead, the artery is dissected free and brought toward the vein. Additionally, akin to the RADAR approach – where the vein is minimally dissected – our technique leaves the vein virtually untouched, thereby preserving its perivascular tissue and nutrient vessels; this, too, sets it apart from conventional side-to-side anastomosis.

Our results demonstrated favorable clinical outcomes by reducing juxta-anastomotic stenosis incidence while improving AVF maturation and patency rates. The modified RADAR group achieved a primary patency rate of 86.05% at 12 months, which is higher than the 72.2% reported by Bai et al. [6] for the RADAR technique in AVF creation in 2022. This superior outcome may be attributed to the fact that our technique maintains the native venous course and preserves the peri-venous supporting tissues, thus achieving vascular stabilization, preventing vessel displacement, and minimizing turbulence formation. Hemodynamic analysis [6,20] revealed that this configuration promotes favorable flow patterns with reduced oscillatory shear, which has been demonstrated implicated in neointimal hyperplasia and subsequent juxta-anastomotic stenosis. Consequently, AVFs created using this technique exhibit superior hemodynamic stability, which accounts for the lower rate of neointimal hyperplasia and, ultimately, the improved long-term patency. Meanwhile, unlike the original RADAR technique, our method preserves the integrity and patency of the radial artery, thus reduces the risks of hand ischemia and permits future AVF or AVG creation at the elbow. The no-touch technique was first proposed for harvesting autologous saphenous veins in coronary artery bypass grafting in 1996 [21], and subsequent studies [22,23] have confirmed improved graft patency. However, no significant benefit of the no-touch technique was observed in our study, a finding consistent with previous reports in AVF creation [4]. This lack of benefit may reflect the fact that certain injuries – such as those induced by vascular clamping, arterial traction, or the anastomosis itself – remain unavoidable despite preservation of the perivenous tissue. Accordingly, complete protection of the venous wall in AVF creation requires not only avoidance of dissection but also optimization of anastomotic geometry and hemodynamic profiles, which represents a key advantage of our modified RADAR technique.

We opted to preserve the distal vein for two reasons. First, previous studies of the RADAR technique consistently demonstrated intact venous integrity without associated swelling or compromised blood flow effects. Second, we believe that preserving the distal vein offers a potentially straightforward and convenient surgical access route for potential future endovascular interventions of the AVF.

There are some limitations in our study. First, this was a single-center study with a limited sample size involving only patients attending our hospital and is therefore not sufficiently representative of the whole population. Second, this is a retrospective study and follow up time is limited. Future multi-center, prospective randomized controlled trial with longer observation time are needed to validate our findings.

## Conclusion

Our modified RADAR technique prevents devascularization of the venous and severing of the radial artery. Compared with the no-touch and conventional techniques, RC-AVF creation by the modified RADAR technique decreases juxta-anastomotic stenosis, improves AVF maturation, and increases primary patency. The method proved feasible, and it should be evaluated in future randomized controlled trials.

## Data Availability

The datasets used or analyzed during the current study are available from the corresponding author on reasonable request.
